# The emerging role of autophagy in the rewarding and stimulant behaviors in models of substance use disorder

**DOI:** 10.3389/fnins.2026.1737046

**Published:** 2026-01-16

**Authors:** America J. Bustos Segura, Troy Gharibani, Maged M. Harraz

**Affiliations:** 1 Department of Psychiatry, University of Maryland School of Medicine, Baltimore, MD, United States; 2 Department of Health Policy and Management, Johns Hopkins Bloomberg School of Public Health, Baltimore, MD, United States; 3 Department of Pharmacology and Physiology, University of Maryland School of Medicine, Baltimore, MD, United States

**Keywords:** autophagy, cannabis, cocaine, morphine, substance use disorder, drug-induced behavior, presynaptic autophagy, postsynaptic autophagy

## Abstract

Substance use disorder (SUD) is a major worldwide health problem with a historic global high of 316 million people using drugs, representing a 15% rise in prevalence over the previous decade. A comprehensive understanding of the pathophysiology of SUD will enable the development of novel therapeutic strategies to improve patient outcomes. Preclinical research on the neurobiology of SUD primarily focuses on the immediate monoaminergic systems response, changes in gene expression, and long-term maladaptive synaptic and circuit-level alterations as the key pathophysiological mechanisms. A few recent publications point to a novel role for the proteostatic process, autophagy, in the rewarding and stimulant effects in animal models of SUD. In this minireview, we summarize the key findings of these reports and discuss potential future directions. These emerging roles expand our understanding of autophagy in the nervous system—from a housekeeping recycling process to a multifunctional regulator of signal transduction, neurotransmission, and behavior—and suggest that autophagy may be a novel therapeutic target in SUD.

## Introduction

Substance use disorder (SUD) is a worldwide major health problem. According to the World Drug Report 2025, 316 million people globally used drugs, with numbers rising faster than population growth, reflecting a 15% rise in prevalence over the preceding decade (World Drug Report 2025, 2025). Substance use disorder starts at the molecular/cellular level, leading to negative behavioral changes (Baldwin et al., 2024). The complexity of addiction lies in the illicit substances’ ability to interact with various cellular targets at different potencies. Hence, illicit substances interact with multiple targets in various neuronal and non-neuronal cell types, leading to negative behavioral and toxic changes. As a result of this complexity, extensive research efforts have been devoted to understanding the various modes of action illicit substances have on signal transduction and neuronal circuitry, leading to behavioral and toxic changes. An in-depth understanding of the neurobiological factors that contribute to the development of substance use disorder is vital for identifying therapeutic targets and creating effective therapeutics.

Autophagy is a proteostatic cellular process that recycles various cellular components, such as proteins and organelles, into lysosomes under conditions such as nutrient deprivation, stress, and trauma (Parzych and Klionsky, 2014). There are three forms of autophagy: macroautophagy, microautophagy, and chaperone-mediated autophagy (Fleming et al., 2022). Macroautophagy is the most studied form of autophagy and referred to hereafter as “autophagy.” Cellular autophagy is induced by autophagy-related genes (ATGs) to form a phagophore that engulfs cytosolic cargo, which then fuses with lysosomes to degrade and recycle basic building components such as amino acids (Mizushima et al., 2011). Autophagy is critical for neuronal survival and has recently been linked to neurodegenerative diseases such as Parkinson’s, Alzheimer’s, Huntington’s, and neuropathies (Mizushima and Levine, 2020; Klionsky et al., 2021b).

Illicit substances modulate autophagy at different potencies, mainly studied as a stress response mechanism or a toxic effect (Wang et al., 2025). In most studies, substance-induced modulation of autophagy is linked to toxic effects, neuroinflammation, oxidative stress, mitochondrial dysfunction, blood–brain barrier dysfunction, and excitotoxicity (Gonçalves et al., 2014). For example, induction of autophagy in microglial cells as a form of cocaine induced neuroinflammation (Guo et al., 2015). However, no studies determined whether cocaine-induced autophagy in microglia mediates the behavioral actions of cocaine. Similarly, most of the studies addressing the role of autophagy in substance use disorder emphasize its role as a stress response and/or a toxic effect of the illicit substance, rather than examining whether autophagy mediates the behavioral actions of the drug relevant to the development of substance use disorder. For more in-depth perspectives regarding the role of autophagy in the toxic effects of illicit substances, the reader is referred to several reviews that focus on the role of autophagy as a stress response/toxic process in substance and alcohol use disorders (Gonçalves et al., 2014; Cao et al., 2017; Jayanthi et al., 2021; Limanaqi et al., 2021; Abdullah et al., 2022; Guo et al., 2023; Bakheet et al., 2025; Malik and Agrewala, 2025; Ruiter-Lopez et al., 2025; Wang et al., 2025; Yu et al., 2025). Recently, a handful of papers have started addressing whether autophagy induced by illicit drugs plays a role in mediating the psychostimulant effects of these drugs. Here, we shed light on this emerging area of research.

## The role of autophagy in the psychostimulant effect of cocaine

Cocaine use disorder is characterized by compulsive use of the drug despite the adverse effects and recurrent relapse following abstinence. The immediate psychostimulant actions of cocaine involve the monoaminergic systems, especially dopamine (DA) (Hall et al., 2004). This initial effect leads to epigenetic-mediated changes in gene expression (Jonkman and Kenny, 2013; Nestler, 2014; Teague and Nestler, 2022), which in turn lead to long-term synaptic and circuit-level changes (Thomas et al., 2008; Siciliano et al., 2015; Wolf, 2016). Decoding the complicated dialog between monoamine regulation, epigenetic regulation of gene expression, and synaptic/circuit level changes will lead to a more comprehensive understanding of cocaine use disorder, paving the way for more effective therapeutic approaches (Wolf, 2010; López et al., 2020). Here, we summarize recent evidence pointing to a novel role for autophagy in the psychostimulant effects of cocaine.

Recent work demonstrated that the autophagy protein Beclin-2 in DA neurons is involved in the rewarding and stimulant effects of cocaine (Kim et al., 2021). Since homozygous deletion of *Becn2/Beclin 2* gene leads to embryonic lethality (He et al., 2013), they used a heterozygous knockout (KO) mouse model and dopaminergic neuron-specific homozygous deletion of *Becn2*. Beclin-2 is an autophagy-related protein that binds to class III PI3K complex (Su M. et al., 2017). Beclin-2 is also part of a class III PI3K-independent form of lysosomal degradation; it helps degrade G-protein-coupled receptors (GPCRs) by binding to GPCR-associated sorting protein 1 (GASP1). GASP1 binds GPCRs and traffics them to the lysosome for degradation after endocytosis (He et al., 2013). Haploinsufficiency of *Becn2* leads to defective autophagy (He et al., 2013) and reduces cocaine-induced DA levels increase in the nucleus accumbens (NAc), hyperlocomotion, conditioned place preference (CPP), and self-administration, suggesting a role for autophagy in the psychostimulant effects of cocaine (Kim et al., 2021). *Becn2* deletion in DA neurons inhibits cocaine-induced hyperlocomotion and CPP, suggesting a primarily presynaptic DA neuron-specific role of autophagy mediating the psychostimulant effects of cocaine. The binding of Beclin-2 to GASP1 may underlie the observed effects of *Becn2* haploinsufficiency on cocaine-induced hyperlocomotion. *Becn2^S97L^* as well as *Becn2* heterozygous KO mice show reduced hyperlocomotion and reduced locomotor sensitization in response to cocaine. Beclin-2-S97L does not bind GASP1. DA receptor 2 (D2R) is a GPCR expressed on the presynaptic terminals of DA neurons and a binding partner of GASP1, which leads to endolysosomal degradation of D2R in the presence of Beclin-2. In the absence of Beclin-2, D2R is not trafficked to the lysosome, increasing its levels in DA nerve terminals, which might at least partially explain the effects of *Becn2* deletion in DA neurons and *Becn2* haploinsufficiency. In support of a role for autophagy in cocaine actions, systemic administration of the upstream autophagy inhibitors SBI-0206965 (ULK-1 inhibitor) and Spautin-1 (destabilizes the Vps34 kinase complex) inhibits cocaine-induced DA levels increase in NAc, hyperlocomotion, and CPP, in WT but not *Becn2* haploinsufficient mice, suggesting that Beclin-2 mediates the effect of autophagy inhibition on cocaine-induced behavior.

Similar work discovered that autophagic degradation of the DA transporter (DAT) contributes to the locomotor stimulant effect of cocaine, implicating autophagy in selective targeting of a membrane protein to regulate cell signaling and modulate cocaine-induced behavior (Harraz et al., 2021). The authors used biochemical and morphologic approaches *in vitro* and *in vivo* to show that cocaine induces neuronal autophagy with extraordinary potency (100 pM levels). Confocal microscopy studies in GFP-LC3 (an autophagy marker) mice show rapid upregulation of GFP-LC3 in tyrosine hydroxylase-positive (TH^+ve^) terminals in NAc 3 min following intraperitoneal injection of 20 mg/kg cocaine. Electron microscopy studies show NAc presynaptic terminals with upregulation of autophagosomes 15 min after stereotaxic injection of 10 femtomoles (sub-nanomolar levels) of cocaine in NAc. Three different pharmacologic autophagy inhibitors reduced cocaine-induced hyperlocomotion; namely, SBI-0206965 (ULK-1 inhibitor), vacuolin-1 (autophagosome-lysosome fusion inhibitor), and hydroxychloroquine (HCQ) (a lysosomal inhibitor). Also, autophagy inhibition by HCQ impairs the expression of cocaine-induced CPP. Though HCQ is a common inhibitor of autophagy, it is important to test additional autophagy inhibitors to exclude off-target effects. In support of this finding, SBI-0206965 (ULK-1 inhibitor) and Spautin-1 (destabilizes the Vps34 kinase complex) inhibit cocaine-induced CPP (Kim et al., 2021), further supporting a role for autophagy in cocaine reward. It is well established that cocaine binds to DAT at high nanomolar to micromolar levels and inhibits DA reuptake, thereby increasing extracellular DA (Hall et al., 2004). Interestingly, two pharmacologic inhibitors of autophagy reduce cocaine-induced increase in DA levels in NAc, suggesting a role for autophagy in the regulation of DA neurotransmission. DAT levels are quickly depleted in NAc synaptosomes 5 min after a 20 mg/kg i.p. injection of cocaine. DAT depletion by cocaine is rescued by two pharmacologic autophagy inhibitors, suggesting that cocaine induces autophagic degradation of DAT in NAc. Despite the potent effect of cocaine inducing autophagy in neurons and depleting DAT in NAc synaptosomes, inhibition of autophagy reduced the rewarding and locomotor stimulant effects of cocaine and inhibited DAT depletion in NAc synaptosomes even when administering high doses of cocaine. Taken together, these findings suggest that at low cocaine levels, autophagy is the predominant mechanism inhibiting DAT through its elimination. At higher cocaine levels both autophagy and direct inhibition by cocaine binding contribute to DAT inhibition. It remains to be elucidated whether autophagy inhibition of DAT by low levels of cocaine is sufficient to mediate the behavioral changes in the absence of direct cocaine inhibition of the transporter.

Using a lentiviral vector to deliver an shRNA against the essential autophagy gene *ATG5*, recent findings show that local inhibition of autophagy in NAc neurons potentiates cocaine-induced hyperlocomotion. Conversely, local activation of autophagy in NAc using rapamycin reduces cocaine-induced hyperlocomotion, suggesting a potential postsynaptic role for autophagy in the regulation of the locomotor stimulant effect of cocaine (Lu et al., 2020). Using biochemical and morphologic approaches as autophagy readouts, high levels of cocaine (10 μM *in vitro* and 15 mg/kg *in vivo*) induce neuronal autophagy in NAc fresh slices. Hence, cocaine (at 10 μM) increases CaMKII, p-AMPK, and LC3II levels. The CaMKII inhibitor KN93 prevents the increase by cocaine of p-AMPK and LC3II levels. SCH23390, an antagonist of DA receptor 1 (D1R), but not sulpiride, an antagonist of D2R, prevents cocaine-induced activation of CaMKII, AMPK, and LC3-II, suggesting that cocaine induces autophagy through D1R in NAc.

It is worth noting that inhibition of cocaine-induced hyperlocomotion is observed following systemic administration of pharmacologic inhibitors of autophagy. The mechanism of action involves presynaptic actions on DAT and D2R (Harraz et al., 2021; Kim et al., 2021). On the other hand, another study showed potentiation of cocaine-induced hyperlocomotion following ATG5 knockdown locally in NAc (Lu et al., 2020). The mechanism of action involves postsynaptic autophagy activation by cocaine through D1R. Hence, it seems that presynaptic autophagy may have the opposite action of postsynaptic autophagy on cocaine-induced hyperlocomotion. Acute systemic administration of pharmacologic autophagy inhibitors, the presynaptic effects of autophagy on cocaine-induced hyperlocomotion seem to outweigh the postsynaptic actions.

## The role of autophagy in the behavioral responses to morphine

Autophagy in DA neurons may be involved in the rewarding, locomotor sensitization, analgesic tolerance, and withdrawal effects of morphine (Su L. Y. et al., 2017). Treating primary midbrain neurons with very high levels of morphine (50–200 μM) induces autophagy in an ATG5 and ATG7-dependent manner. The increase in autophagy induced by morphine is inhibited by naloxone, suggesting that morphine induces autophagy through the μ-opioid receptor 1 (OPRM1) (Su et al., 2017a). It is important to mention here that these levels of morphine are highly toxic and were previously detected in the brain of mice following acute fatal dose of 300 mg/kg (Guillot et al., 2007). Hence, the induction of autophagy by 50–200 μM morphine might reflect a cytotoxic effect. *In vivo*, mice with deletion of the *ATG5* or *ATG7* genes in TH^+ve^ neurons since birth show reduced morphine-induced CPP and locomotor sensitization, impaired antinociceptive tolerance in response to morphine, and amelioration of morphine withdrawal symptoms (Su et al., 2017a). These findings implicate autophagy in mediating the psychostimulant effects of morphine. Interestingly, deletion of the ATG5 or ATG7 genes in TH^+ve^ neurons from birth has previously been shown to affect the biology of DA neurons. Hence, mice with deletion of the *ATG7* gene in DA neurons since birth exhibited unusually large dopaminergic axonal profiles, released higher amounts of DA upon stimulation, and demonstrated quicker recovery at the presynaptic level (Hernandez et al., 2012). These long-term changes might complicate the conclusion implicating autophagy in mediating the behavioral effects of morphine (Su et al., 2017a). An acute strategy in inhibiting autophagy is required to confirm these conclusions. In this context, locomotor sensitization following repeated morphine administration is reduced by acute inhibition of autophagy using intracerebroventricular injection of the autophagy inhibitor 3-methyladenine (Su et al., 2017a). Conversely, locomotor sensitization to morphine is increased by intracerebroventricular injection of the autophagy activator rapamycin, suggesting a role for autophagy in mediating morphine-induced locomotor sensitization (Su et al., 2017a).

Deletion of the autophagy-related gene *Sirt1* in D1R expressing neurons since birth reduced heroin-induced CPP and hyperlocomotion in mice (Huang et al., 2024). The NAD-dependent protein deacetylase sirtuin-1 (encoded by the *Sirt1* gene) forms a molecular complex with many essential autophagy proteins such as ATG5, ATG7, and ATG8. Deletion of Sirt1 in mice leads to defective autophagy resembling ATG5 KO mice leading to perinatal mortality (Lee et al., 2008). Hence, the experimental results reported by Huang et al. should be interpreted cautiously. Further characterization of the D1R-expressing neurons in the *Sirt1* conditional KO mice is required to better interpret these findings.

## The role of autophagy in the behavioral responses to cannabis

Recent work shows that cannabinoid-induced impairment of motor coordination involves inhibition of striatonigral autophagy (Blázquez et al., 2020). Δ9-tetrahydrocannabinol (THC) inhibits autophagy selectively in the striatum *in vivo* and in primary striatal neurons *in vitro*. THC impairs motor coordination as assessed by the rotarod test but does not affect the performance in the open field test (Blázquez et al., 2020). The FDA-approved selective mTOR inhibitor temsirolimus disinhibits autophagy (Choi et al., 2012). Temsirolimus rescues the THC-induced impairment of motor coordination and striatal autophagy *in vivo* (Blázquez et al., 2020). Similarly, the natural disaccharide and autophagy activator trehalose rescues the THC-induced impairment of motor coordination and striatal autophagy *in vivo* (Blázquez et al., 2020).

Further, deleting the cannabinoid receptor 1 (CB1R) in the medium spiny neurons (MSN) in the striatonigral pathway rescues the THC-induced impairment of motor coordination and striatal autophagy *in vivo.* On the other hand, deleting CB1R in glutamatergic neurons’ outflow onto MSNs did not change the THC-induced impairment of motor coordination and striatal autophagy *in vivo* (Blázquez et al., 2020). Taken together, these findings suggest that CB1R expressed on D1R-MSNs, but not on corticostriatal projections, mediate the THC-induced impairment of motor coordination and striatal autophagy *in vivo*. Inhibition of mTORC1 by overexpressing dominant negative raptor selectively in striatal D1R MSNs rescues the THC-induced impairment of motor coordination *in vivo* (Blázquez et al., 2020), suggesting that disinhibition of autophagy in D1R MSNs prevents THC-induced impairment of motor coordination.

## Cross-substance comparisons

**Figure 1:**
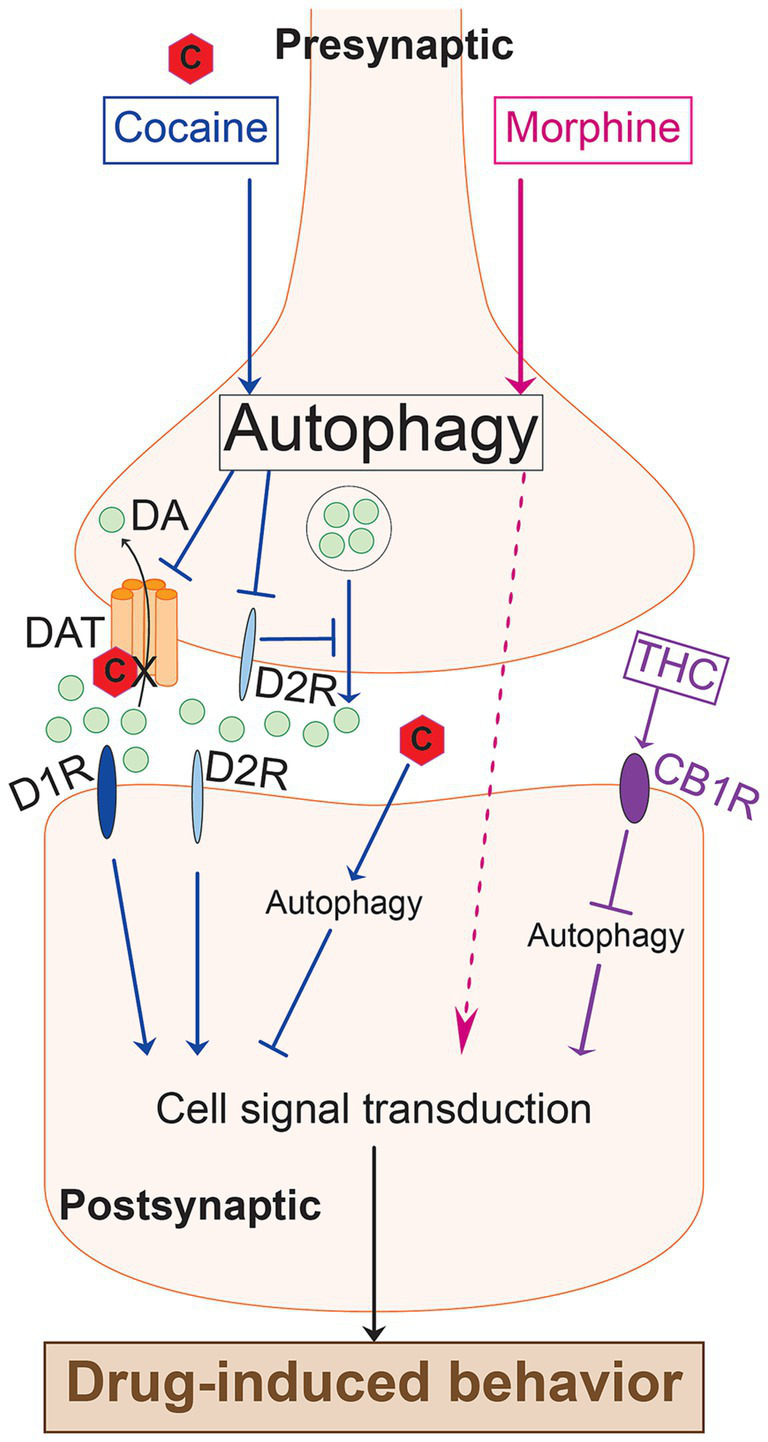
A diagram illustrating cross-substance comparisons of the role of autophagy mediating drug-induced behavior. Cocaine induces presynaptic autophagy in DA neurons. Autophagy, in turn, targets DAT, inhibiting its function and increasing extracellular DA. Cocaine binds to DAT and inhibits it, leading to increased extracellular DA. Cocaine-induced presynaptic autophagy inhibits D2R activity, which in turn disinhibits DA release from the presynaptic terminal, leading to increased extracellular DA. The increased dopamine on the postsynaptic side acts through D1R and D2R to activate signal transduction and mediate the drug-induced behavior. Morphine induces presynaptic autophagy in DA neurons. Autophagy, in turn, activates signal transduction in the postsynaptic neuron, thereby altering behavior. The dotted arrow depicts an unknown mechanism. Cocaine induces postsynaptic autophagy, which inhibits signal transduction that leads to the drug-induced behavior. THC inhibits postsynaptic autophagy via the CB1 receptor. Autophagy on the postsynaptic side activates signaling pathways that lead to drug-induced behavior.

Presynaptic autophagy in DA neurons mediates cocaine and morphine hyperlocomotion and their rewarding effects (Figure 1) (Su et al., 2017a; Harraz et al., 2021; Kim et al., 2021). On the other hand, blocking postsynaptic autophagy in the NAc potentiates cocaine-induced hyperlocomotion and rewarding effects (Figure 1) (Lu et al., 2020). Hence, postsynaptic autophagy action seems to be opposite to the presynaptic autophagy in the case of cocaine. However, systemic inhibition of autophagy reduces cocaine-induced hyperlocomotion and reward (Harraz et al., 2021; Kim et al., 2021). This suggests that presynaptic autophagy outweighs postsynaptic effects on cocaine-induced hyperlocomotion and reward.

Like the postsynaptic autophagy in the NAc, which opposes the cocaine-induced behaviors, cannabinoid-induced impairment of motor coordination involves striatonigral autophagy inhibition (Figure 1) (Blázquez et al., 2020). These observations might indicate emerging themes regarding the effects of presynaptic vs. postsynaptic autophagy roles in drug-induced behavior. However, the studies addressing these questions are still limited in number and confined to short-term exposure to the drugs. Further research is needed to interrogate whether these emerging themes are consistent and whether they extend into long-term drug use endpoints such as extinction and relapse.

## Concluding remarks and future directions

The role of autophagy in mediating the rewarding and stimulant effects in substance use disorder is an emerging field. Only a handful of research studies have been performed so far. However, the evidence for an important role of autophagy in SUD is strong. Since autophagy is a recycling process, it is critical to limit its manipulations to short-term approaches, especially in neurons. Due to the postmitotic nature and the elaborate network of dendrites, dendritic spines, synapses, and axon arborizations, neurons have high energy and high maintenance demands. Hence, long-term disruption of autophagy by genetic or pharmacologic approaches leads to neurodegenerative changes (Hara et al., 2006; Komatsu et al., 2006), which could contribute to the observed experimental outcomes, complicating the interpretation of results, whether due to autophagy disruption or degenerative changes. Acute manipulation of autophagy, on the other hand, minimizes these compounding factors (Klionsky et al., 2021a).

The papers discussed in this minireview focused on neurons and mainly on short term drug administration. It will be interesting to learn whether autophagy in various glial cells plays a role in the rewarding and/or stimulant effects of illicit drugs. Further, the role of autophagy in long-term drug use is still unknown. Specifically, it will be important to investigate the role of autophagy in drug extinction, cravings, and relapse. Also, the molecular mechanism linking illicit drug exposure to the autophagy pathway machinery is still unknown. Discovering how illicit drugs regulate autophagy and how autophagy, in turn, contributes to the maladaptive changes in SUD will expand our understanding of the pathophysiology of the disease. Specifically, it will be interesting to know whether there’s a crosstalk between autophagy modulation by illicit drugs and the monoaminergic systems, epigenetic regulation of gene expression, synaptic, and circuit-level changes in SUD. Cocaine-induced autophagy regulates dopaminergic neurotransmission, likely, through DAT and D2R (Harraz et al., 2021; Kim et al., 2021). Questions remain about what other proteins and cellular processes do drug-induced autophagy regulate contributing to the pathophysiology of SUD. Recent studies implicate autophagy in the regulation of synaptic plasticity (Nikoletopoulou et al., 2017; Chang et al., 2024; Smith et al., 2025; Thakur and O’Connor-Giles, 2025). It will be interesting to find out whether autophagy regulation by illicit drugs contributes to the synaptic/circuit-level maladaptive changes in SUD.

Traditionally, autophagy has been viewed as a housekeeping process that helps maintain homeostasis by removing aged and damaged proteins/organelles to support neuronal survival. Recent evidence implicates autophagy in the selective targeting of proteins in response to neuronal activity, thereby regulating neurotransmission, synaptic plasticity, and behavior.
